# Operative experience on descending aorta with Takayasu Arteritis: a review

**DOI:** 10.3389/fcvm.2023.1181285

**Published:** 2023-06-21

**Authors:** Yining Fu, Yuexin Chen

**Affiliations:** Department of Vascular Surgery, Peking Union Medical College Hospital, Beijing, China

**Keywords:** surgery, descending aorta, takayasu arteritis, bypass suegery, endovascular treatment

## Abstract

Patients with Takayasu arteritis (TA) and descending aorta involvement often experience insidious onset and slow progression, leading to irreversible vascular lesions despite medication therapy. Surgical management plays a crucial role in resolving hemodynamic disturbances and has shown promise in improving the outcomes of this patient population, owing to significant advancements in surgical expertise. However, studies focusing on this rare disease are lacking. This review summarizes the characteristics of patients with stenosis in descending aorta, emphasizing surgical approaches, perioperative management, and disease outcomes. The operative approach depends on lesion location and extent. Existing studies have confirmed that the choice of surgical modality significantly influences postoperative complications and long-term prognosis in patients, highlighting the effectiveness of bypass surgery as a favorable option in clinical practice with a satisfactory long-term patency rate. To mitigate postoperative complications, it is advisable to conduct regular imaging follow-ups to prevent the deterioration of the condition. Notably, the occurrence of restenosis and pseudoaneurysm formation deserves particular attention due to their impact on patient survival. The use of perioperative medication remains a topic of debate, as previous studies have presented divergent perspectives. The primary objective of this review is to provide a comprehensive perspective on surgical treatment and offer customized surgical approaches for patients in this population.

## Introduction

Takayasu arteritis (TA) is a nonspecific inflammatory disease of the aorta and its main branches, causing a range of arterial stenosis/occlusion or dilatation. Previous studies revealed that stenosis (93%) is the most frequent vascular presentation, and the abdominal aorta is the most frequent lesion location in the Asian population (1, 2). Patients with stenosis in the descending aorta (including the thoracic and abdominal aorta) may present life-threatening complications before 40, causing poor prognosis (3, 4). Surgical treatments are required in 18%–70% of all TA patients, with a substantial proportion experiencing stenosis in the descending aorta (5, 6).

Bypass surgery has been associated with a good long-term patency rate but is complex and requires a multidisciplinary approach. Endovascular therapy is less invasive and reproducible, but its patency rate is inferior to the former. Indeed, each method has pros and cons, which necessitates the tailored surgery design. It is imperative to evaluate each patient's condition individually, assess the surgical benefit and risks, and choose the appropriate surgical approach. A comprehensive evaluation of large systemic vessels is necessary to determine the optimal surgical approach. The utilization of advanced technology can assist in developing a precise surgical plan. In our center, for complex cases, we employ hemodynamic simulation to calculate pressures at various anatomical sites, identify optimal anastomosis locations, and re-evaluate the pressure to assess the effectiveness of the planned surgery. This approach allows us to validate the efficacy of the surgical intervention.

This article aims to present a comprehensive overview of current practices in the management of patients with descending aorta involvement. We will summarize the findings of previous studies, explore the impact of different surgical approaches on prognosis, and propose optimized management strategies for this specific patient population. We advocate that perioperative treatment and surgical modalities will continue to advance, offering hope and improved outcomes for these patients.

## Methods

Considering the rarity of Takayasu arteritis, a comprehensive search was conducted to ensure that the objective reflects clinical practice. The study selection specifically focused on a population that underwent surgery targeting the descending aorta, which includes the thoracic and/or abdominal aorta. The research encompassed retrospective case-control analyses, case series, and case reports (Table 1). Studies that analyzed the outcomes of TA patients with multiple lesions, including thoracic and/or abdominal aortic stenosis, but were not exclusively focused on that specific aspect were marked "Null" in Table 1.

**Table 1:** Summary of surgery.

First author	Case	Surgery	Number	Follow-up	Patency rate	Survival rate	Complications	Reference
Miyata, T	32	Bypass Aortic patch	28 4	19.8 y	100%	Null	17 anastomotic aneurysms	(7)
Joh, J.-H.	2	Bypass Endarterectomy	1 1	4–75 m	100%	100%	1 migraine-like headache	(8)
Saadoun, D.	31	Surgery Endovascular treatment	18 13	6.5 y	Null	Null	Null	(9)
Lee, G.	14	Bypass Endovascular Treatment	5 9	3.2 y	100% 85.7%	100%	1 chylothorax, 1 aortic dissection and 1 aortic rupture	(10)
Lee, B.-B.	3	Endovascular treatment	3	46.8 m	Null	Null	Null	(11)
Labarca, C.	7	Bypass Endovascular treatment	2 5	10 y	Null	Null	Null	(6)
Kim, S. M.	10	Bypass Patch	9 1	60m	100%	100%	Null	(12)
Hinojosa, C. A.	2	Bypass	1 1	81m 46 m	100% 100%	100% 100%	Stenosis at aortic anastomosis 1 year after surgery	(13)
Setty, H. S. N.	10	Endovascular treatment	10	Null	Null	Null	Null	(14)
Hinojosa, C. A.	4	Bypass Aortic tube graft Endovascular treatment	2 1 1	Null	Null	Null	Null Recurrence at 11 months	(15)
Che, W.	48	Endovascular treatment	48	12 m	A total of 5 (10.9%) patients developed in-stent restenosis, which were resolved by reintervention (restenting in 3 patients and reangioplasty alone in 2 patients)	100%	1 patient developed retroperitoneal hemorrhage and one developed flow-limiting dissection that involved bilateral renal arteries perioperatively	(16)
Rosa Neto, N. S.	6	Bypass Endovascular treatment	4 2	Null	Null	100%	Null	(17)
Kim, Y. S.	9	Bypass	9	36.3 m	100%	5-year survival was 79 ± 13%.	3 patients with chylothorax, 1 patient with a wound problem, and 1 patient with bleeding due to pancreatic injury and mediastinitis	(18)
Diao, Y.	28	Bypass Endovascular treatment	11 17	48.5 ± 38.5 m	Null	Null	Null	(19)
Fan, L.	15	Endovascular treatment	15	2.88 y	Null	100%	thoracic aorta (*n* = 5, 13.9%), abdominal aorta (*n* = 2, 5.6%)	(20)
Joseph, G.	397	Endovascular treatment	397	34 m	98%	100%	Dissection 6.8% Rupture/PsA 3.3%	(21)

Null, the details of the data are not shown in the manuscripts.

## Diagnostic criteria

The diagnostic criteria for Takayasu arteritis established by the American College of Rheumatology (ACR) in 1990 are widely accepted (22). However, these criteria were developed using a small sample size, limiting their generalizability and independent validation, impacting their applicability in clinical practice. In 1995, a modification to the diagnostic criteria was proposed, eliminating age restrictions. This modification resulted in an increased diagnostic sensitivity (92.5%) and specificity (95.0%) (23, 24). The most recent classification criteria for Takayasu arteritis developed jointly by the American College of Rheumatology (ACR) and the European League Against Rheumatism (EULAR) in 2022 have shown superior performance compared to the previous criteria. The 2022 criteria demonstrated a sensitivity of 93.8% and a specificity of 99.2%. These criteria were developed using a cohort of 316 TA patients and further validated using an independent dataset comprising an additional 146 TA patients from an international cohort (25). Notably, the 2022 criteria emphasized the importance of clinical symptoms, vascular physical examination findings, and vascular imaging in the classification of the disease. These criteria exhibited excellent performance across patients from different regions.

## Demographics and angiographic patterns

Ascending aorta and aortic arch involvement is more commonly observed in patients from East Asia, while South Asian patients tend to exhibit a higher prevalence of abdominal aorta and renal artery involvement, and among Mexican patients, Numano V disease is the most frequently encountered subtype (26–28). Besides, gender plays a role in the distribution of vascular involvement in TA (29). In terms of vascular involvement in Takayasu arteritis, females are more commonly affected by thoracic aorta involvement, while males tend to have a higher susceptibility to abdominal aorta involvement (30, 31). Specifically, lesions in the abdominal aorta are diffusely distributed, with approximately 69% occurring in the suprarenal region, 23% in the juxtarenal region, and 8% in the infrarenal aorta (27). The Numano angiographic classification is widely utilized to categorize TA patients; however, it exhibits limitations in differentiating patients based on clinical presentation and formulating appropriate treatment plans (32). In this review, we focus on patients with descending aorta involvement (including Numano IIb, III, IV, V) as they often display similar clinical presentations and require similar surgical and medical approaches.

## Signs and symptoms

The clinical presentation of Takayasu arteritis varies depending on the specific lesions involved. In patients with stenosis in the descending aorta, symptoms can arise from hypertension proximal to the aortic stenosis or hypotension distal to it. If the lower abdominal aorta is affected, claudication may be observed. Involvement of the suprarenal or juxtarenal aorta can lead to impaired renal perfusion and subsequent renal hypertension. Stenosis of the thoracic aorta can result in hypertension due to increased workload on the heart. Notably, hypertension is the most common symptom in TA patients with descending aorta involved with a prevalence of 60%–100%, probably associated with renal hypoperfusion or ischemia, stenosis of the descending aorta, severe aortic regurgitation, and reduced aortic compliance (12, 27, 33). If untreated, most patients die before 35 due to the complications of uncontrolled hypertension (3). Lower extremity claudication is the second most common symptom, presenting in 15%–50% of patients (4, 33). Other symptoms, including headache and syncope, can be observed in these patients (33). The formation of aneurysms (24%) is not rare in TA patients, and >50% of TA patients may develop aneurysms in the course of the disease. More importantly, multiple synchronous lesions (stenotic and aneurysmatic) may coexist in the same patients (34).

Hypertension, being the most prevalent symptom in Takayasu arteritis patients with descending aorta involvement, holds significant value as an indicator for assessing disease control and prognosis. However, the involvement of upper limb arteries may result in inaccurate blood pressure measurements, leading to delayed diagnosis and poor prognosis. For patients without bilateral upper limb arteries involved, the higher value from the arms is recorded; for patients with unilateral upper limb artery affected, the reading from the unaffected side is used; for patients with stenosis in bilateral upper limb arteries, the central blood pressure is collected to reflect the core blood pressure (35).

## Assessment of disease activity

The definition of active disease in TA is based on the National Institutes of Health (NIH) guidelines (36, 37). Current acute-phase reactants used to assess disease activity include erythrocyte sedimentation rate (ESR) and C-reactive protein (CRP). Elevated ESR is one of the strongest indicators of disease progression (15). However, it is important to note that vascular damage can progress without systemic inflammation. Current evidence suggests that 30%–40% of patients may appear clinically stable (in quiescence) but can still be confirmed to be in the active phase based on surgical histopathology findings (5, 33, 38).

[18F] Fluorodeoxyglucose combined positron emission and computed tomography (18F-FDG-PET-CT) and magnetic resonance imaging (MRI) can assess arterial inflammation by measuring the degree of vessel wall edema. These imaging examinations can confirm vascular wall inflammation, especially in patients with normal levels of inflammation markers (38, 39).

It should be borne in mind that 18F-FDG-PET-CT cannot accurately distinguish arteritis from metabolically active vascular remodeling due to the lack of inflammatory cell selectivity. However, new means of imaging examination have emerged to address this limitation. One such approach involves targeting macrophage activation, as macrophages play a significant role in inflammatory infiltrates. The somatostatin receptor subtype-2 (SST2), expressed on activated macrophages, has been identified as a biomarker for vasculitis. A recent study demonstrated that SST2 positron emission tomography (PET)/magnetic resonance imaging (MRI) showed potential in defining disease activity in TA patients with a more sensitive and accurate diagnosis (40).

Contrast-enhanced ultrasound (CEUS) is also a promising approach to assessing disease activity. A previous study showed that the severe stenosis depicted by CEUS in the carotid artery wall was correlated with vascular inflammation detected by PET/CT (41–44). Given the convenience of CEUS imaging, we introduced CEUS imaging as a routine in surveillance protocol.

## Imaging

Imaging assessment primarily focuses on the aorta and its major branches in diagnosing Takayasu arteritis. While various imaging modalities are available, angiography remains the cornerstone for diagnosing TA. Computed tomographic arteriography (CTA), magnetic resonance angiography (MRA), and digital subtraction angiography (DSA) are the most frequently preoperative study used to define anatomy, which can facilitate the assessment of the extent and severity of the arterial injury (45). CTA is the imaging modality of choice for diagnosing and monitoring Takayasu arteritis in nearly 60% of patients. The widespread availability, cost-effectiveness, and superior image resolution compared to MRA account for the popularity of CTA. Its accessibility, affordability, and ability to provide detailed and high-quality images make CTA an invaluable tool in the evaluation and management of TA patients during both the diagnostic and follow-up stages.

Doppler ultrasonography (Doppler US) is also used to quantify the severity of luminal narrowing as a less invasive approach. Reports indicate that among TA patients who received imaging assessment, 58.8% underwent CTA, while 29.9% underwent MRA, and Doppler ultrasonography was used in 11.3% of all patients (46).

Current evidence suggests that 18F-FDG-PET-CT facilitates early diagnosis in 7% of patients and may improve prognosis (15, 47). PET-CT has also become a diagnostic test for assessing arterial inflammation and monitoring the response to immunomodulatory therapy (6, 48). Repeat PET-CT should be considered to confirm disease activity during this period.

The EULAR 2018 guidelines suggest that if the patient presents with recurrent or new symptoms, regular imaging assessment is needed during follow-up (49). Among these imaging methods, MRI is the most frequently used for follow-up because it avoids the use of radiation (46).

## Cardiovascular manifestations

As the most common symptom, hypertension is one of the most valued indicators to assess disease control and prognosis. However, the involvement of upper limb arteries may result in inaccurate blood pressure measurements, leading to delayed diagnosis and poor prognosis.

Heart involvement is not rare in TA patients, emphasizing the need for conducting electrocardiograms (ECGs). It is now understood that congestive heart failure induced by stenosis lesions in descending aorta is the main cause of death in patients with Takayasu arteritis (50). Another symptom, aortic regurgitation, is present in 13% to 44% of cases due to increased afterload on the heart (51, 52). Thus, ECG is recommended as a routine examination in all TA patients to reflect valvular and atrioventricular abnormalities. The ejection fraction, the aortic regurgitation, the diameter of the ascending aorta, the diameter of the aortic sinus, the aortic valve annular diameter, and the left ventricular end-diastolic diameter have been reported as indicators in previous studies (53, 54). ECG can also be used for follow-up examination since surgery can mitigate TA-related hypertension and relieve left ventricular hypertrophy (18).

Coronary involvement is a commonly observed lesion in patients with Takayasu arteritis. When there is suspicion of coronary stenosis, state-of-the-art CT coronary angiography has emerged as a reliable non-invasive method (55, 56). This imaging technique offers high isotropic spatial resolution ranging from 0.23 mm to 0.35 mm, while maintaining a low radiation dose profile. CT coronary angiography provides detailed visualization of the coronary arteries, aiding in the assessment of coronary stenosis in TA patients with accuracy and precision.

## Management

### Surgical treatment

Severe stenosis, defined as a narrowing of 70% or more, can result in hemodynamic disturbances that lead to symptomatic end-organ ischemia. In such cases, surgical interventions are crucial in addressing stenosis in the descending aorta and its visceral artery branches. The indications for TA patients with descending aorta involvement mainly include refractory hypertension, cardiac ischemia, aortic regurgitation, and extremity claudication. In order to optimize patient outcomes, preoperative blood pressure control is recommended, aiming to maintain blood pressure within the normal range. However, if hypertension is primarily caused by a significant narrowing of the descending aorta, surgical intervention is preferred regardless of the patient's hypertension status. While it is generally advisable to avoid surgery during the acute phase of the disease, if patients are in a critical condition, surgery becomes necessary (17).

Aortic stenosis can often involve adjacent visceral arteries, with the splanchnic and renal arteries being common coexisting lesions (27). About 80% of patients with abdominal aortic stenosis also exhibit renal artery stenoses (27, 57). Renal reconstructions are usually performed in patients with decreased renal function or refractory renovascular hypertension. Mesenteric reconstructions are conducted in patients with abdominal pain or other related symptoms. However, although more than 50% of patients present with splanchnic occlusive lesions, only 6% experienced symptomatic bowel ischemia, suggesting prophylactic treatment is required to improve splanchnic stenosis (27).

**Figure 1:**
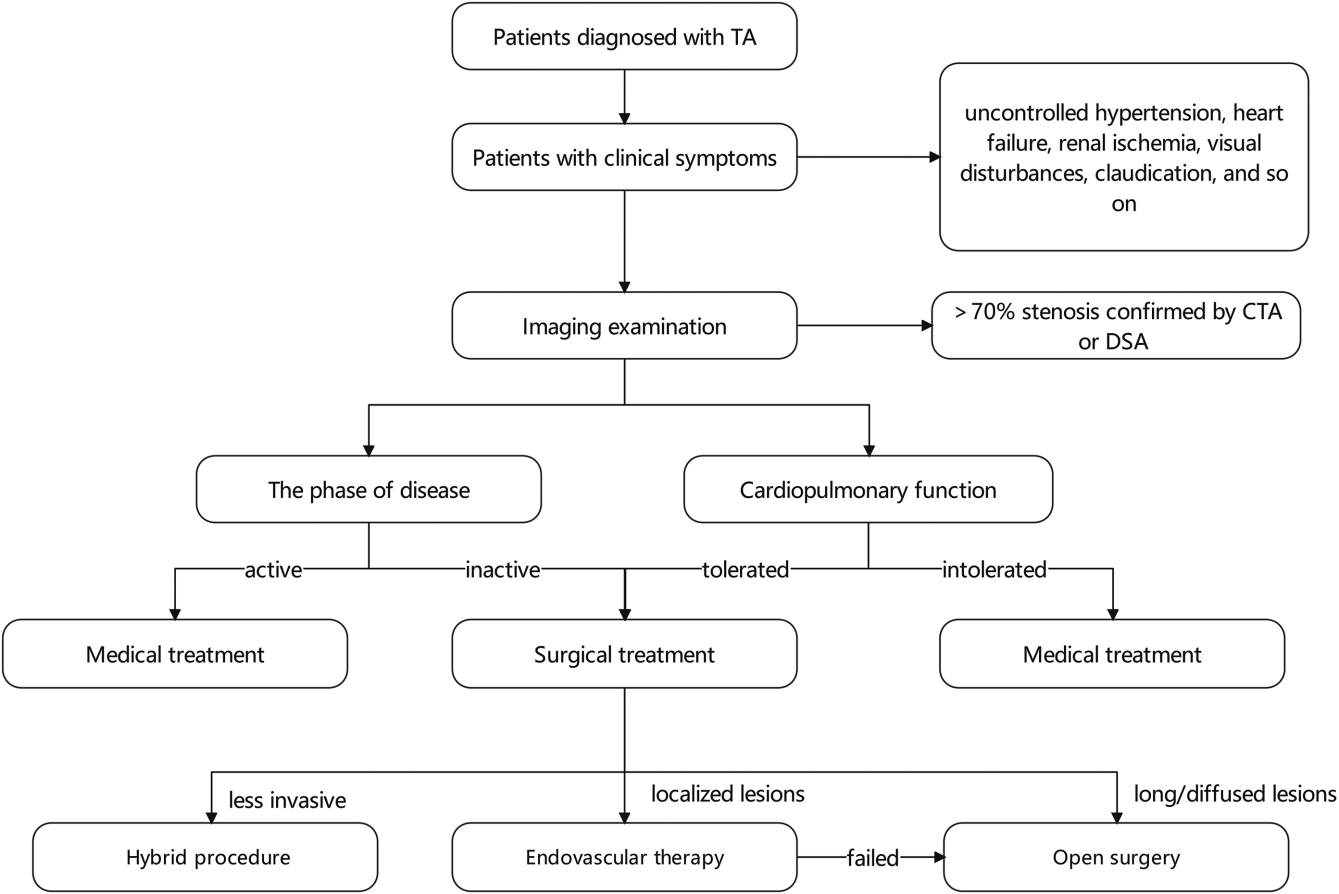
The treatment algorithm of TA patients.

The location and extent of the lesion should be taken into account during the selection of the surgical procedure. To date, no standard therapy is applicable to all patients. Methods for aortic reconstruction include bypass surgery, interposition graft, patch angioplasty, and endovascular therapy (12). Bypass procedures may be favored in patients having too extensive coarctation segments or complex lesions (15). In other cases with short coarctation, arterial patch, interposition graft, and endovascular therapy may be attractive (58) (Figure 1).

Endovascular technologies (including angioplasty and stent-graft repair) are suitable for localized stenoses distant from the renal, celiac, and superior mesenteric arteries. These minimally invasive procedures are advantageous for young patients since they can be repeated and may obviate the need for open surgery. In particular, endovascular interventions allow luminal dilation in children with a developing aorta without interfering with the vessels. The first percutaneous transluminal angioplasty(PTA) for a patient with abdominal aortic coarctation was performed in 1983 (59). However, due to the relatively high failure rate, balloon angioplasty alone is less effective than stent-graft repair (60, 61). It is widely recognized that endovascular therapy has certain inherent limitations. For instance, the arterial wall may become weakened following balloon angioplasty, resulting in aneurysm formation in 5% to 20% of cases (62, 63). Furthermore, the elastic fibers disruption in the media vessel wall and vascular fibrosis in the adventitia contribute to poor patient response to both endovascular therapies, leading to restenosis rates in 25% to 60% of cases (58, 64–66). Moreover, vessel stiffness can limit endovascular therapy's effectiveness, resulting in under-dilatation and risk of stent graft rupture (67).

Aorta endarterectomy is indicated for young patients with short and isolated segment (4, 8, 68). Butcher and his colleagues first reported a satisfactory result in cases with aortoiliac arterial occlusion treated by endarterectomy (69). However, endarterectomy is not recommended for TA patients. Theoretically, aortic endarterectomy addresses only the intimal fibrosis, while fibrosis in the adventitial or periadventitial layers may persist, posing a challenge to treatment efficacy (27). While it may offer the possibility of a thoracoabdominal bypass in the later stages of life, it is widely considered a standby option.

Considering that the TA patients are relatively young, long-term durability is a vital factor in choosing surgical approaches, leading to bypass surgery as the most frequent choice in TA with descending aorta involvement (26). This approach can resolve extensive arteriopathy by an ingenious surgical design, such as using a sequential bypass to re-establish the blood supply of the ischemic organs. Some studies also revealed that the need for open surgery remains, even when percutaneous procedures presented with an increasing and widening ambit (21). To date, several techniques for extra-anatomic bypass have been proposed.

The left posterolateral thoracotomy enables the procedure to be performed on the ascending aorta, aortic arch, and entire descending aorta. However, some studies have raised concerns about potential complications, including massive bleeding, paraplegia, and chylothorax (70–72). Median sternotomy was first reported by Vijayanagar R. et al. (73) in 1980. The benefits and disadvantages of this surgical modality have been established. Optimal operative exposure for the entire descending aorta can be achieved. It also allows simultaneous cardiac procedures or reoperation to be conducted. However, the requirement of both thoracotomy and laparotomy poses a great challenge to patients' cardiorespiratory functions, making it more appropriate for younger patients. For elderly patients, aorta-femoral bypass is a better option to augment the vascular bed and retrograde renal blood flow with a lower risk of damaging the collateral circulation.

Considering the chronic inflammatory state of patients, open surgery can be overly invasive. However, there is evidence supporting the use of hybrid aortic repair as a more favorable option in a less invasive way for complex lesions. This approach involves bypassing the supra-aortic vessels or debranching the visceral or renal arteries before performing stent grafting on the aortic arch and descending aorta. Joseph G. et al. (21) proposed that about 80% patients treated with surgical procedures underwent also endovascular procedures. Hybrid aortic repair offers advantages such as shorter operation time, reduced surgical complexity, and increased success rates. It is particularly beneficial for patients with a heavily calcified aorta (74–76).

Regarding the choice of the anastomotic site, the utilization of supra celiac bare area for distal anastomosis was first reported by Wukasch D. C. et al. in 1977, which featured reduced bleeding and decreased incidence of complications due to the short course of the graft (77, 78). However, it should be noted that this less invasive approach may have certain drawbacks. For instance, inadequate exposure may be an issue in patients with abdominal obesity or barrel-shaped thorax, and managing bleeding from the distal anastomosis may be challenging. As an alternative, an ascending-to-infrarenal abdominal aortic bypass may be considered. Although longer grafts increase the risk of complications from adjacent organs, the long midline incision facilitates exposure of the whole length of the descending aorta, making anastomosis and hemostasis easier (79). More importantly, the ascending-to-infrarenal abdominal aortic bypass can also provide significant antegrade flow to permit optimal renal perfusion, which relieves renovascular hypertension (4). According to the current literature, the double-suture aortic anastomotic technique is applied to prevent postoperative anastomotic aneurysms (80).

A statistically significant difference in restenosis rates has been reported between different graft materials. Polytetrafluoroethylene (PTFE) grafts demonstrated a superior patency rate to Dacron grafts at a 7-year follow-up (100% vs. 58%, *P* = 0.005) (58). The graft diameter was consistent with the mean diameter of descending aorta. A 14 mm–16 mm graft was deemed sufficient for most women, while an average man required a 16 mm–18 mm graft for adequate perfusion (27). Notably, oversized grafts compared to the aorta have been recommended in children to accommodate future growth (4, 27, 64).

### Perioperative medications

Corticosteroids are generally the first-line treatment to control the disease activity, and cytotoxic drugs are added for those patients with disease progression on steroid therapy (64). However, the optimal timing for initiating immunosuppressive therapy upon confirmation of the diagnosis remains a subject of debate. Hinojosa C. A. et al. (15) believed that administering the immunosuppressive drugs as early as possible could arrest disease progression and reverse early clinical symptoms. Perera and colleagues proposed a similar finding that immunosuppression before the endovascular intervention significantly improved results (*P* = 0.001) (45). In contrast, Young Su Kim and co-workers formulated that fibrosis and calcification are predominantly disease-specific alterations rather than vascular wall inflammation for those in the chronic inactive phase, which means using immunosuppressive agents such as cytotoxic agents or steroids is unnecessary (18).

Similarly, no consensus has been reached on the efficacy of postoperative medication use. Some authors postulated that the restenosis rate is lower with post-surgical immunosuppressive treatment, while others argued that there were no differences among patients treated with or without corticosteroids (5, 81, 82). A previous study indicated that for patients on medication therapy, 93% in the open surgery group and 86% in the interventional procedure group exhibited good long-term patency (45). However, other studies showed there was no difference between groups (47, 83).

Previous studies reached an agreement regarding antiplatelet agent use since the TA-related hypercoagulable state can lead to arterial ischemic events, and patients can benefit from anticoagulant therapy (39, 84).

In recent years, the pathogenesis of TA has been better understood, which has led to the development of targeted biotherapies aimed at inhibiting signaling pathways. Recent studies have shown promising results for biological disease-modifying agents (bDMARDs), such as TNF-α inhibitors and IL-6 inhibitors, as well as targeted synthetic disease-modifying agents (tsDMARDs), such as JAK inhibitors75–80. Although limited evidence exists for some bDMARDs, such as Rituximab, Abatacep, and Ustekinumab, they also require further investigation (85–87).

## Complications and prognosis

The prognosis of TA is heavily influenced by the presence or severity of complications. A study from the late 1980s, conducted at a time when diagnostic imaging and medical treatments were less advanced, found that most patients with descending aorta involvement would not survive past the age of 35 years (3). It has been established that graft-related complications, including restenosis and anastomotic aneurysms, are the most common complications after surgery (88). Other study also proposed that peri-interventional dual antiplatelet therapy, concurrent surgery, and technical failure were predictors for complications (*P* < 0.05) (20).

Several studies have revealed that the most common complication in both open and endovascular groups is restenosis, and patients who underwent endovascular procedures showed a higher rate of restenosis (*P* < .001). The patency rate of surgical bypass varied from 64 to 100%, while that of PTA ranged from 29% to 83% (5, 7, 58, 82, 89–91). Consistently, our prior retrospective study, which examined 116 TA patients who received surgery or endovascular interventions (such as PTA and stent-graft repair), revealed that both surgical approaches were effective and safe. However, open surgical repair was found to be more suitable for complex lesions due to its longer durability (19). Moreover, other factors are related to restenoses, such as hypertension (*P* = 0.01), dyslipidemia (*P* = 0.01), and high-dose steroids (*P* = 0.012) (6).

It has been reported that pseudoaneurysms at the anastomotic site occur with an incidence of 12.2%, 21.2%, and 37.3% in the 10-year, 20-year, and 30-year follow-ups, respectively (12). This complication probably results from hypertension and the degradation of graft materials (92). Notably, most patients presented with no symptoms or signs and were detected incidentally, which led to devastating results. Therefore, even though the patients showed no signs of anastomotic aneurysms, regular follow-ups are needed (27).

It is widely thought that postoperative complications are associated with disease activity. However, the association between postoperative complications and disease activity has been controversial. Kim, S. M. et al. (12) thought disease activity could affect outcomes and long-term survival. However, Fields C. E. et al. (5) found that long-term survival was not affected by disease activity, supported by findings reported by Weaver F. A. et al. (93).

Studies reported that the overall survival rate at 20 years was 62.3%–73.5% (7, 12) and death was mainly attributed to cardiovascular events (7, 50). Among these, the incidence of congestive heart failure-induced death ranged from 3% to 40% (7, 10). Further investigation also revealed that the risk factors for heart failure include pulmonary hypertension, aortic valve or coronary artery involvement, onset age >38 years, and serum tumor necrosis factor (TNF)-*α* concentration >10 pg/ml (54).

Ishikawa K. et al. (94) identified four predictors for mortality risk factors: complications (retinopathy, secondary hypertension, aortic regurgitation, and aneurysmal formation), the pattern of the clinical course, age, and year of diagnosis. Other studies confirmed that postoperative hypertension (*P* = 0.028), type of disease (*P* = 0.0142), age at operation (*P* = 0.0052), and presence of an aneurysmal lesion (*P* = 0.0106) were significantly associated with postoperative events and survival rate (7, 33).

## Discussion

The need for multiple vascular surgeries involving both endovascular and surgical procedures is not uncommon in TA patients due to the prolonged duration of the disease. Table 1 presents several studies highlighting the surgical management of descending aorta stenosis associated with TA. Most patients benefit from the correction of abnormal hemodynamics and the relief of hypertension. Current evidence suggests that 74%–90% of patients experience improvement in hypertension-related symptoms after the surgery (3, 27, 33). Surgery also played a role in relieving left ventricular burden. A study revealed that almost all patients demonstrated improved cardiac function, with some cases showing significant enhancements in interventricular septal diameter (IVSD, *P* = 0.016) and left ventricular mass index (LVMI, *P* = 0.017) (18). An updated retrospective study from our research team also demonstrated that surgery could significantly improve the prognosis of patients.

Taketani T. et al. (33) reported that after surgical treatment, the overall survival and event-free survival rate were 62.3% and 58.4% at 20-year follow-up, and postoperative hypertension was a significant predictor of event-free survival (*P* = 0.028). Kalangos A. et al. (58) demonstrated the safety and effect of the surgery, with hypertension being controlled and cardiac function returning to normal postoperatively. Stanley J. C. et al. (27) assessed the outcomes of different operative treatments in patients with abdominal aortic coarctation (4 were diagnosed with inflammatory aortitis) and found that more than 90% of patients benefit from surgery. The above studies overlap in their assertion that surgery is safe and effective for TA in all arterial areas. Herein, we focused on patients with descending aorta involvement and comprehensively analyzed the surgical methods and clinical outcomes of this patient population. We aimed to review the relevant literature in detail and summarize the unique characteristics of these patients.

With significant inroads achieved in surgical techniques, multidisciplinary decision-making, targeted biotherapies, and comprehensive postoperative monitoring and treatment, surgeries can be performed with low morbidity and improved quality of life (58). In addition, a deeper understanding of the pathophysiology of arterial reconstruction in TA patients helps to reduce surgical complications.

Preoperative evaluation is critical in guiding surgical decisions regarding the method and timing of interventions in TA patients. Despite their relatively young age, TA patients often present with severe cardiac, renal, and pulmonary complications due to the insidious nature of the disease and its atypical clinical manifestations. The involvement of multiple arterial bifurcations further adds to the complexity of the condition. As a result, comprehensive medical evaluations are essential prior to surgery. These evaluations encompass various aspects, such as assessing vascular lesions, determining disease activity, evaluating cardiopulmonary function, and overall disease status. While erythrocyte sedimentation rate (ESR) and C-reactive protein (CRP) are commonly used as acute-phase indicators in TA, it should be noted that these serum markers may not always accurately reflect vascular wall inflammation. In fact, approximately 30%–40% of patients in the active phase of the disease may exhibit normal ESR and/or CRP levels (26). More importantly, combining clinical presentation, serum markers, and imaging examinations such as PET-CT and MRI is crucial for accurately assessing disease activity in TA patients, especially when acute-phase reactants exhibit poor sensitivity during periods of low disease activity.

The presence of nonspecific symptoms often leads to delayed diagnosis of TA, resulting in disease progression and the occurrence of ischemic events. Early diagnosis and prompt treatment are essential in order to improve the prognosis of patients with TA (95).

Besides, although the indications for surgery have not been definitively established, we advocate the safety and efficacy of surgical intervention. Prior to 1988, the average life expectancy of patients with atypical TA was only 35 years, likely due to less advanced and effective diagnostic techniques that led to delayed treatment (96). Kalangos A. et al. (58) revealed that patients couldundergo reconstructive procedures with satisfactory midterm and long-term outcomes regardless of the extent and severity of vascular lesions. We also demonstrated in another article that surgical revascularization is superior in relieving symptoms and improving the prognosis compared to conservative treatment. Moreover, given that these patients are complicated with coexisting renal and splanchnic artery occlusion, surgery aims to restore the renal and splanchnic artery flow based on the symptoms.

More importantly, the inconsistency of findings may be attributed to different follow-up duration. It has long been thought that at least a 20-year follow-up is mandatory to reflect the impact of surgical therapy since about 10% of patients were treated with secondary surgeries in the late stages of follow-up (5, 27). However, the debate regarding the choice of surgical options remains unsettled due to the limited number of studies with long-term follow-up, varying prognoses among patients, and inconsistent durations of follow-up.

It should be borne in mind that TA is a rare disease, and it is challenging to obtain a large cohort of patients undergoing surgical treatment. Further research is required to confirm the efficacy and safety of these procedures, which will enable us to offer improved treatment options for patients with TA.

## Conclusion

The ongoing advancements in surgical techniques establish surgery as a viable treatment option for TA. While more studies are required to establish definitive criteria for surgical indications, existing data indicate that most patients can benefit from surgery.
