# Corrigendum: Operative experience on descending aorta with Takayasu arteritis: a review

**DOI:** 10.3389/fcvm.2024.1525748

**Published:** 2024-12-11

**Authors:** Fu Yining, Yuexin Chen

**Affiliations:** Department of Vascular Surgery, Peking Union Medical College Hospital, Beijing, China

**Keywords:** surgery, descending aorta, Takayasu arteritis, bypass surgery, endovascular treatment

A Corrigendum on Operative experience on descending aorta with Takayasu arteritis: a review By Fu Y, Chen Y. (2023). Front. Cardiovasc. Med. 10:1181285. doi: 10.3389/fcvm.2023.1181285

In the published article, there was an error in the Funding statement. The funding statement was displayed as “This project was supported by the National High Level Hospital Clinical Research Funding”. The correct Funding statement appears below.

**FUNDING**

This project was supported by the National High Level Hospital Clinical Research Funding (2022-PUMCH-A-191).

The authors apologize for this error and state that this does not change the scientific conclusions of the article in any way. The original article has been updated.

